# Inflammatory Pseudotumor of the Mesentery

**DOI:** 10.5334/jbsr.2586

**Published:** 2021-10-14

**Authors:** Jonas De Melio, Veerle Mertens, Stefaan Gryspeerdt

**Affiliations:** 1 AZ Delta, BE

**Keywords:** Abdominal radiology, IPT, inflammatory pseudotumor, epithelioid variant, mesentery

## Abstract

**Teaching point**: An inflammatory pseudotumor can occur almost everywhere in the body and has nonspecific imaging findings.

## Case history

**Figure 1:**
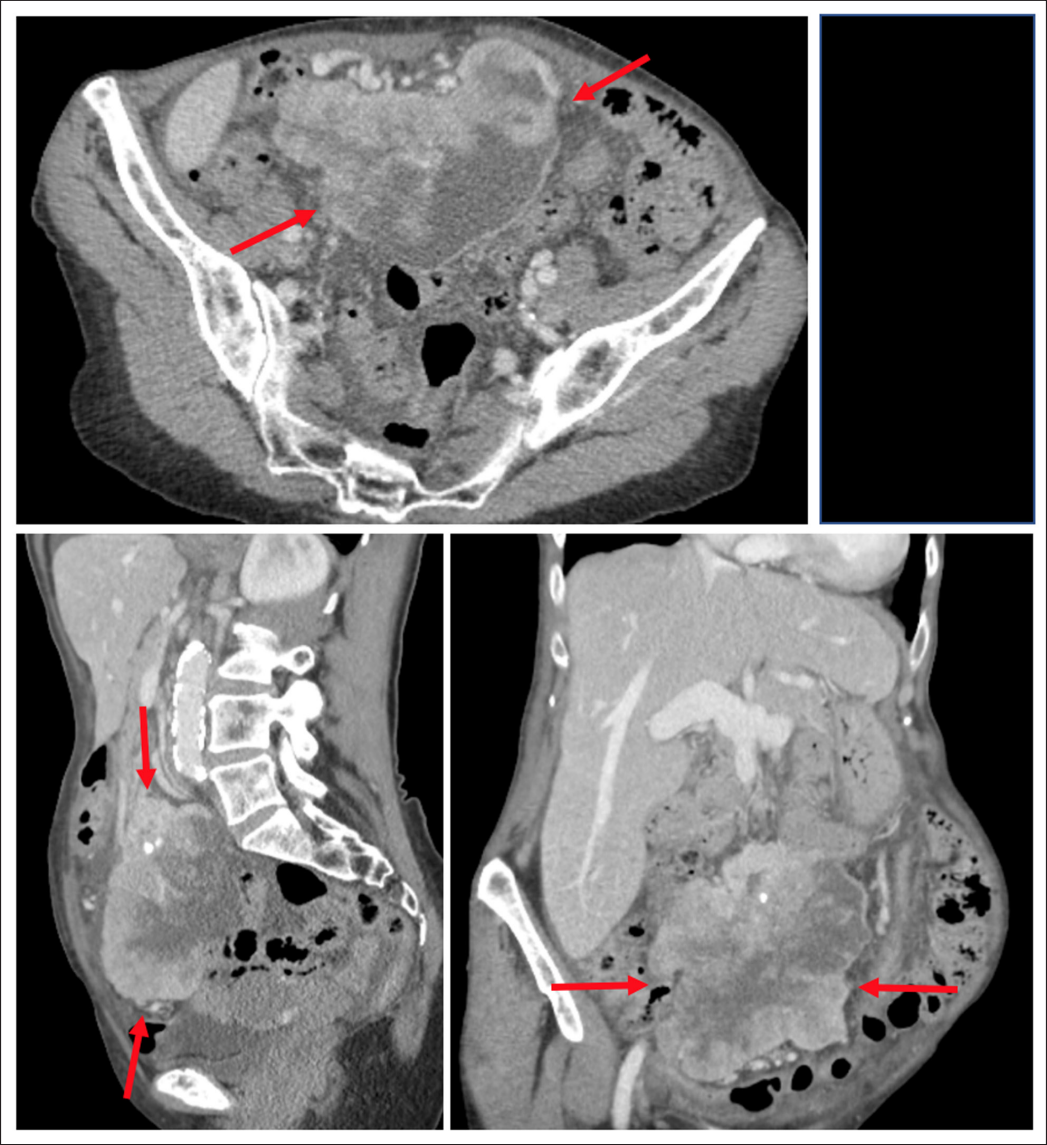


**Figure 2:**
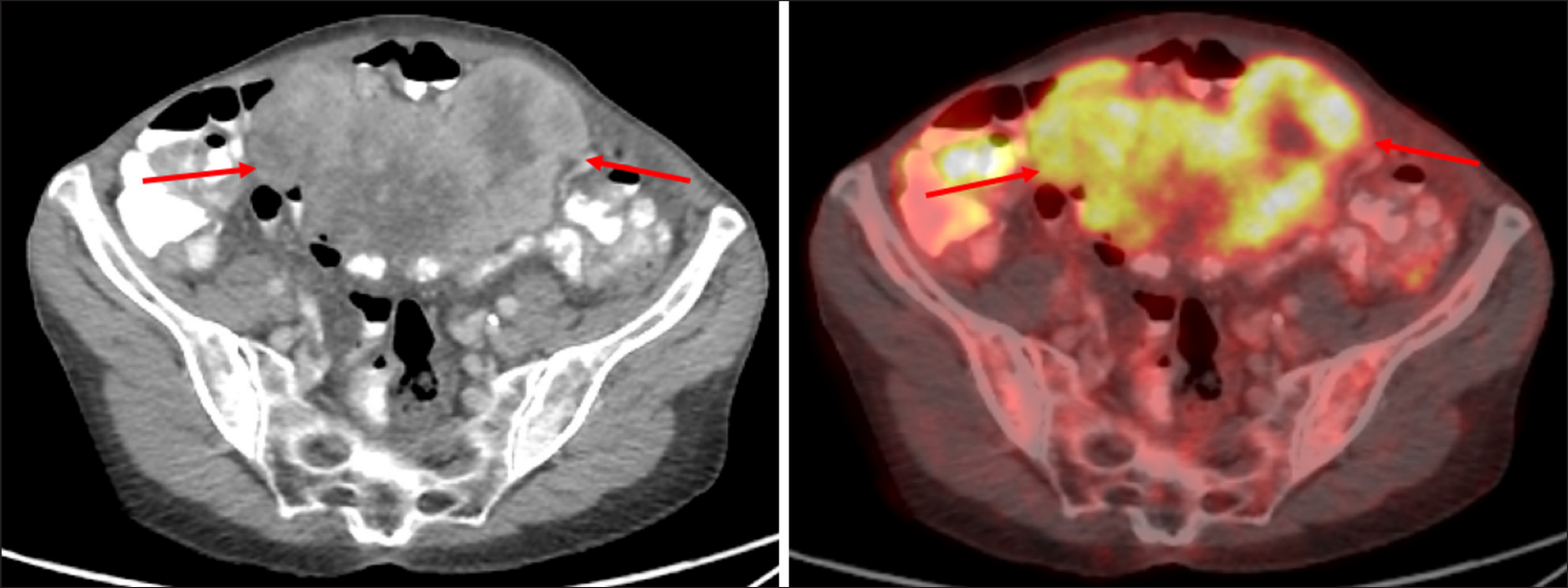


**Figure 3:**
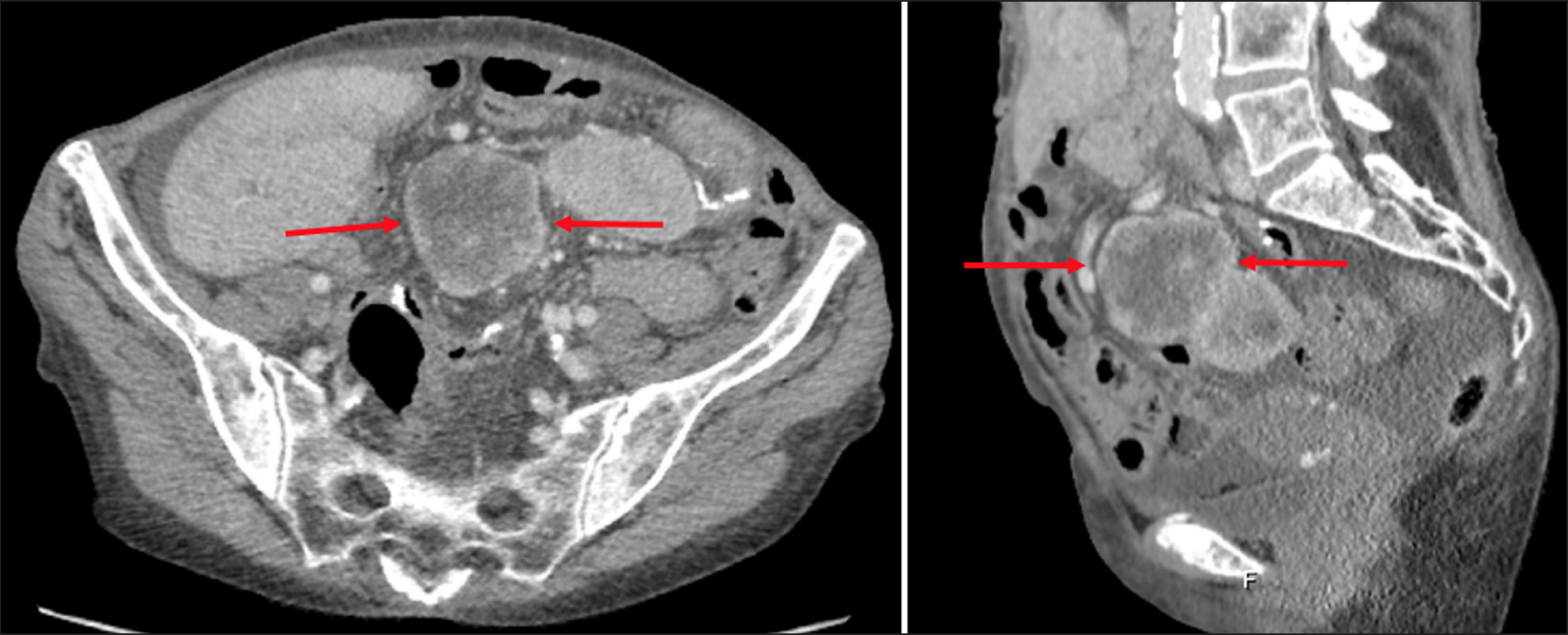


We present the case of a 73-year-old woman, referred to the gynecologist for the workup of a mass found in the lower abdominal region during clinical examination. Subsequent vaginal ultrasound was suggestive of a mass in the pelvic cavity. A body computed tomography (CT) confirmed the presence of a heterogeneously enhancing, partially ill-defined lesion in the lower abdomen with dimensions of 12 × 9 × 11 cm (***Figure 1***, arrows). The lesion was located on the mesentery and contained multiple central areas of non-enhancement, suggesting necrosis. Some small central intralesional calcifications were also present. Additional 18-Fluoro-Deoxy-Glucose positron emission tomography – CT showed enhanced metabolism of the lesion (***Figure 2***, arrows). No lesion suspicious for metastasis was present. An ultrasound-guided biopsy was performed, and the histopathological examination made the diagnosis of an epithelioid variant of an inflammatory myofibroblastic tumor. Laparotomy with resection of the tumor followed, which confirmed the mesenteric origin of the inflammatory myofibroblastic tumor. There was tumor invasion of the right hemicolon and terminal ileum, which were also resected during surgery. Follow-up CT three months later, revealed extensive local tumor recurrence at the level of the mesentery with associated diffuse liver metastasis and ascites (***Figure 3***, arrows). Palliative treatment was initiated.

## Comment

An inflammatory pseudotumor (IPT) is composed of cells involved in acute and chronic inflammatory processes. Inflammatory myofibroblastic tumor is a term, which is sometimes used instead of IPT, due to the presence of myofibroblasts and histiocytes in the lesion. IPT’s can present as a single mass or multiple masses and most often occur in the orbit or lung. Nevertheless, IPT’s can be found almost everywhere in the body. A mesenteric IPT, as in our case, is an uncommon manifestation, especially since this entity tends to occur more in young adults or children, in contrast to our 73-year-old patient. A mesenteric IPT can have well-defined boundaries or an ill-defined infiltrative margin with possible associated invasion of adjacent bowel loops. The enhancement pattern can be variable, with homogeneous or heterogeneous enhancement. Associated central calcifications and areas of necrosis can be present. These non-specific and variable imaging characteristics make it difficult to differentiate from other disease entities, such as lymphoma or soft-tissue sarcomas. Finally, the clinician should provide a higher rate of surveillance for patients with an epithelioid variant of intra-abdominal IPT. These epithelioid variants of intra-abdominal IPT’s often have a more aggressive behavior, with a faster recurrence rate [1].
